# Effect of COVID-19 Lockdown on Small Animal Trauma Patterns in Australia: A Multicentre Study

**DOI:** 10.3389/fvets.2022.908679

**Published:** 2022-06-01

**Authors:** Mara C. Hickey, Elizabeth Napier, Hui Mei Ong

**Affiliations:** ^1^Sydney School of Veterinary Science, Faculty of Science, The University of Sydney, Sydney, NSW, Australia; ^2^Animal Referral & Emergency Centre, Broadmeadow, NSW, Australia; ^3^Melbourne Veterinary School, Faculty of Veterinary and Agricultural Sciences, University of Melbourne, Werribee, VIC, Australia

**Keywords:** trauma, COVID, cats, dogs, financial effect

## Abstract

**Objective:**

To determine whether patterns of trauma changed following the start of local lockdowns due to COVID-19.

**Design:**

Multi-institutional retrospective study assessing patients presenting within 2 years prior to local lockdown due to COVID-19 and 1 year following lockdown inclusive.

**Setting:**

Two university teaching hospitals and one private referral center in Australia.

**Animals:**

Dogs and cats with a presenting complaint of known or suspected trauma.

**Interventions:**

Patient signalment, date of presentation, trauma type, treatment interventions and patient outcome (survival to discharge, cardiopulmonary arrest, or euthanasia) were recorded in a web-based data capture system (REDCap).

**Measurements and Main Results:**

Three thousand one hundred eighty-nine patients (682 cats and 2,507 dogs) were included in the study. Overall trauma prevalence was 2.9% with pre-lockdown prevalence of 2.8% and post-lockdown prevalence of 3.1% (*p* < 0.001). Cats had higher rates of blunt trauma while penetrating trauma was more prevalent in dogs (*p* < 0.001). Juvenile patients were also more likely to have blunt trauma when compared to adult patients (*p* < 0.001). Patient age and sex characteristics did not differ when comparing the 2 time periods. Compared to pre-lockdown, blunt and penetrating trauma patterns changed post-lockdown in dogs and cats (*p* < 0.001 for both). Interventions were performed more frequently (*p* = 0.039) in the post-lockdown with surgical procedures having a significant increase (*p* = 0.015). Survival rates increased post-lockdown for both species (*p* < 0.001) with financially driven euthanasia being less common than in pre-lockdown for dogs (*p* = 0.02).

**Conclusions:**

Trauma patterns changed for cats and dogs in the post-lockdown period. Compared to pre-lockdown, trauma prevalence was higher with a decrease in mortality rate. No increase in juvenile patients was identified post-lockdown. A decrease in financially driven euthanasia and an increase in interventions suggest no negative financial effect from COVID-19 lockdown on trauma patient care in Australia.

## Introduction

Trauma patterns in cats and dogs have been reported multiple times with some differences in the types of trauma dependent on species, age, sex/reproductive status and regional population differences (urban vs. suburban vs. rural) (1–8). Cats most commonly present with blunt trauma while dogs more frequently present with penetrating trauma, with head trauma more likely leading to death in cats when compared to dogs (1–7). Trauma occurs in a higher proportion of male cats and dogs than female ones, and younger animals are more likely to experience traumatic injury than older animals (4, 8).

The coronavirus (COVID-19, hereafter referred to a COVID) pandemic (9) and subsequent lockdowns have had a significant impact on the lifestyle and regular day to day activities of Australians (10) and those around the globe (11) since March 2020. In Australia, government-mandated lockdowns (12) were controlled at the regional or municipal level with mildly different levels of movement restriction. Overall, the population was confined to their homes. Only essential workers were allowed to travel locally for work; all others were permitted out of the home for exercise (including walking the dog), to seek medical care, and to obtain food and necessities.

Data from human studies have identified a reduction in the presentation of trauma patients during lockdown periods in Australia with the Royal Adelaide Hospital and Westmead Hospital Sydney reporting decreases of 33% and 23–34%, respectively (13, 14). Notably, there was a decrease in road traffic accidents, likely due to reduced movement and vehicle transport. With changes in human-pet interactions (with owners home with their pets more of the time) and increased interactions between dogs (with owners walking them more frequently to leave the confines of home), we hypothesized that the prevalence of trauma and trauma patterns for cats and dogs would differ when comparing pre-lockdown periods (BEFORE) with periods during lockdown (AFTER).

Increases in pet ownership have been definitively reported for Australia during the pandemic, with an increase of 20–45% in adoptions (15), leading to 69% of Australian households owning pets (16). With social media identifying a “puppy boom” and anecdotal reports of increased adoption of kittens, we hypothesized that the ages of patients presenting for traumatic injuries would be lower in the AFTER period compared to the BEFORE.

Finally, due to reports of changes in income (17) due to job losses, we hypothesized that expensive interventions such as oxygen administration, blood transfusions, surgical procedures and mechanical ventilation would be performed at different rates BEFORE vs. AFTER, also leading to different rates of euthanasia being performed for financial reasons.

## Materials and Methods

Electronic medical records of the University Veterinary Teaching Hospital Sydney (SYD), the University of Melbourne (MEL) and the Animal Referral & Emergency Centre (AREC) were reviewed. Cases were identified from the 3-year study period using one of two ways: by querying records for the term “trauma” in the history text or by searching the hospital's emergency case log. The study period consisted of two subsets: a pre-lockdown period when each hospital was conducting normal business functions and no limits were placed on human activities, and a post-lockdown period when local public health orders restricted human activities and each hospital ceased entry of clients into the building (curb-side service). The start of curb-side service for each hospital was defined as the date of lockdown and was the first day of the 1-year post-lockdown period. The pre-lockdown time period was a 2-year period ending the day before the date of lockdown. The date of lockdown varied between hospitals based on local public health orders.

Exclusion criteria included any of the following diagnoses: identification of a fish hook on or in the body, corneal abrasion, lameness due to a misstep, torn nail, abscess, self-trauma, or bandage-related trauma. Incomplete medical records were a reason for exclusion, but patients were not excluded from the study if their age or sex was unrecorded. Patients that presented dead on arrival were included in the study.

Information collected included the hospital location, date of visit, patient signalment (species, age, sex), trauma type (blunt, penetrating, unknown or polytrauma), interventions (blood transfusion, oxygen administration, surgical procedure, mechanical ventilation), patient outcome (survival to discharge or death), the cause of death [cardiopulmonary arrest (CPA) vs. euthanasia], and the reason for euthanasia if performed (financial only, poor prognosis only, both financial and prognosis, other, and unknown if the reason could not be identified from the medical record). The behavior leading to the trauma event was not reported as this was not commonly recorded in the medical record. As one of the goals of this study was to identify if there were any financial repercussions from the lockdown, only interventions associated with high cost were identified; minor wound care, medication administration and IV fluids, for example, were not included. Three of the four major interventions were included as they are the primary interventions recorded in the VetCOT trauma registry (5) and oxygen administration was chosen as the fourth intervention as the cost for this increases hospitalization fees by 50% in the participating hospitals, indicating a major financial impact.

Data were collected with a web-based data capture system (REDCap) (18).

## Statistical Methods

Data were visually inspected, and calculations of skewness & kurtosis were performed to evaluate for normality. When continuous data were assessed for normality, no normally distributed data were identified. These data were therefore reported as the median with an interquartile range (IQR). Categorical data were reported as frequency or proportion. Trauma prevalence was calculated as the mean prevalence for all participating hospitals. Patients were grouped into time categories [BEFORE (pre-lockdown) and AFTER (post-lockdown)], age categories [juvenile (<1 year) or adult (≥1 year)] and species categories (feline or canine), and rates of interventions and outcome were assessed between categories using Pearson's chi-square and Fischer's Exact Test. A *p*-value < 0.05 was considered significant. Data were analyzed using IBM SPSS Statistics v24 (IBM 2016. IBM SPSS Statistics for Windows, Version 24.0. IBM).

## Results

### Overall Patient Population

A total of 3,189 patients fulfilled inclusion criteria with 415 from SYD, 1,287 from MEL and 1,487 from AREC. Table 1 shows data for cats and dogs over the three-year period of the study. Cats comprised 21.4% of all patients enrolled in the study, and dogs, 78.6%. The median age of all species was 4 years of age (IQR 7), with 20% of all patients categorized as juvenile. The majority of patients were male animals (53.3%), followed by female animals (46.3%) with 11 patients (0.4%) having no recorded sex. The number of desexed patients were approximately double that of non-desexed patients (66.2% of males and 69.0% of females reported as desexed).

**Table 1:** Patient characteristics, interventions, and outcomes over the 3-year study period.

	**Count (%) or median (IQR)**
**Total count**	**3,189 (100%)**
**Location**
AREC	1,487 (46.6%)
MEL	1,287 (40.4%)
SYD	415 (13%)
**Patient signalment**
**Species**
Feline	682 (21.4%)
Canine	2,507 (78.6%)
Age	4 (7)
**Age group**
Juvenile	635 (19.9%)
Adult	2,554 (80.1%)
**Sex & desexing status**
Male entire	572 (17.9%)
Male desexed	1,126 (35.3%)
Male unreported	2 (0.06%)
Female entire	449 (14.1%)
Female desexed	1,021 (32%)
Female unreported	8 (0.3%)
Unknown	11 (0.3%)
**Trauma type**
Penetrating	*1,684 (52.8%)*
Blunt	*1,340 (42%)*
Unknown	*118 (3.7%)*
Polytrauma	*47 (1.5%)*
**Trauma subtypes**
** Penetrating subtypes**	**Count (% total trauma)**
Bite	1,114 (34.9%)
Impalement	59 (1.9%)
Laceration	475 (14.9%)
Non-accidental	13 (0.4%)
Unknown	27 (0.8%)
Other	4 (0.1%)
** Blunt subtypes**	**Count (% total trauma)**
HBC	650 (20.4%)
Fall	296 (9.3%)
Crush	168 (5.3%)
Ran into object	50 (1.6%)
Struck by object	40 (1.3%)
Non-accidental	13 (0.4%)
Unknown	102 (3.2%)
Hanging	10 (0.3%)
Other	10 (0.3%)
**Interventions**
None	2,447 (76.7%)
Oxygen	505 (15.8%)
Transfusion	423 (13.3%)
Surgical procedure	586 (18.4%)
Mechanical ventilation	4 (0.1%)
**Outcome**
Survival	2,841 (89.1%)
Euthanasia	239 (7.5%)
Cardiopulmonary arrest	109 (3.4%)
**Reason for euthanasia**
Poor prognosis	127 (53.1%)
Financial	58 (24.3%)
Both financial & prognosis	51 (21.3%)
Unreported	38 (15.9%)
Other	2 (0.8%)

*Adult, patients ≥ 1 year of age; AREC, Animal Referral & Emergency Centre; HBC, hit by vehicle; Juvenile, patients < 1 year of age; MEL, University of Melbourne Veterinary Hospital; SYD, University Veterinary Teaching Hospital Sydney*.

Penetrating trauma was most commonly reported (52.8%), followed by blunt trauma (42%), unknown trauma (3.7%), and polytrauma (1.5%). Hit by vehicle (HBC) was the most common cause of blunt injury at 48.5% and bites were the most common cause of penetrating injury at 66.2%.

For 76.7% of patients, no interventions were performed. Oxygen supplementation was administered to 15.8% of patients, 13.3% of patients received one or more blood transfusions, 18.4% of patients underwent a surgical procedure, and mechanical ventilation was performed on 0.13% of patients. A number of patients had more than one intervention.

Most patients survived (89.1%) with 7.5% of all patients euthanized and 3.4% dying from cardiopulmonary arrest. Poor prognosis was the most commonly cited reason for euthanasia (53.1%).

The overall trauma prevalence was 2.9%.

### Before vs. After

Lockdown at SYD started on 23/03/2020, at AREC on 16/03/2020, and at MEL on 25/03/2020. Total patients enrolled BEFORE numbered 1,917 and 1,272 patients were enrolled AFTER.

Table 2 shows data according to time period. There was no significant difference in the proportions of cats and dogs, median age, sex distribution and neuter status in the two periods.

**Table 2:** Patient characteristics, interventions, and outcomes by time period.

**Time period:**	**BEFORE**	**AFTER**	

			Significance^*^
	**Count (% of total)**		**(Values**
	**or median (IQR)**		***≤ 0.05 are in bold***
			***italics*)**
**Total count**	**1,917**	**1,272**	
Location			*P* = 0.264
AREC	908 (47.4%)	579 (45.5%)	
SYD	235 (12.2%)	180 (14.2%)	
MEL	774 (40.4%)	513 (40.3%)	
**Patient signalment**
Species			*P* = 0.595
Feline	416 (21.3%)	266 (20.9%)	
Canine	1,510 (78.7%)	1,006 (79.1%)	
Age	4 (7)	4 (6)	*P* = 0.085
Age group			*P* = 0.113
Juvenile	359 (18.7%)	276 (21.7%)	
Adult	1,520 (79.3%)	993 (78.2%)	
Sex & desexing status			*P* = 0.138
Male entire	350 (18.3%)	222 (17.5%)	
Male desexed	689 (35.9%)	437 (34.4%)	
Male unreported	0 (0%)	2 (0.2%)	
Female entire	270 (14.1%)	179 (14.1%)	
Female desexed	598 (31.2%)	423 (33.3%)	
Female unreported	2 (0.1%)	6 (0.5%)	
Unknown	8 (0.4%)	3 (0.2%)	
**Trauma type**			***P < 0.001***
Penetrating	1,057 (55.1%)	627 (49.3%)	
Blunt	804 (41.9%)	536 (42.1%)	
Unknown	44 (2.3%)	74 (5.8%)	
Polytrauma	12 (0.6%)	35 (2.8%)	
**Trauma Subtypes**
** Penetrating subtypes**	**Count (% total trauma)**		***P < 0.001***
Bite	711 (37.1%)	403 (31.7%)	
Impalement	45 (2.3%)	14 (1.1%)	
Laceration	280 (14.6%)	195 (15.3%)	
Non-accidental	2 (0.01%)	11 (0.9%)	
Unknown	16 (0.8%)	11 (0.9%)	
Other	4 (0.2%)	0 (0%)	
** Blunt subtypes**	**Count (% total trauma)**		***P < 0.001***
HBC	432 (22.5%)	218 (17.1%)	
Fall	148 (7.7%)	148 (11.6%)	
Crush	100 (5.2%)	68 (5.3%)	
Ran into object	29 (1.5%)	21 (1.7%)	
Struck by object	22 (1.1%)	18 (1.4%)	
Non-accidental	7 (0.4%)	6 (0.5%)	
Unknown	54 (2.8%)	48 (3.8%)	
Hanging	8 (0.4%)	2 (0.2%)	
Other	3 (0.02%)	7 (0.6%)	
**Interventions**
None	1,490 (77.7%)	957 (75.2%)	***P = 0.038***
Oxygen	293 (15.3%)	212 (16.7%)	*P* = 0.299
Transfusion	239 (12.5%)	184 (14.5%)	*P* = 0.110
Surgical procedure	326 (17%)	260 (20.4%)	***P = 0.015***
Mechanical ventilation	2 (0.1%)	2 (0.02%)	*P* = 0.523
**Outcome**			***P = 0.013***
Survival	1,683 (87.8%)	1,158 (91%)	
Mortality	234 (12.2%)	114 (9%)	
Euthanasia	158 (8.2%)	81 (6.4%)	
Cardiopulmonary arrest	76 (4%)	33 (3.6%)	
**Reason for euthanasia**	**Reported as count**		*P* = 0.091
	**(% of total euthanasia)**		
Poor prognosis	87 (55.1%)	40 (49.4%)	
Financial	41 (25.9%)	17 (21%)	
Both financial & prognosis	37 (23.4%)	14 (17.3%)	
Unreported	18 (11.4%)	20 (24.7%)	
Other	1 (0.6%)	1 (1.2%)	

*Adult, patients ≥ 1 year of age; AFTER, 1 year period including the first day of local hospital lockdown; AREC, Animal Referral & Emergency Centre; BEFORE, 2-year time period prior to local hospital lockdown; HBC, hit by vehicle; Juvenile, patients < 1 year of age; MEL, University of Melbourne Veterinary Hospital; Overall, entire 3 year of study; SYD, University Veterinary Teaching Hospital Sydney*.

* *Significance from Chi Square or Fischer's Exact Test*.

**Figure 1:**
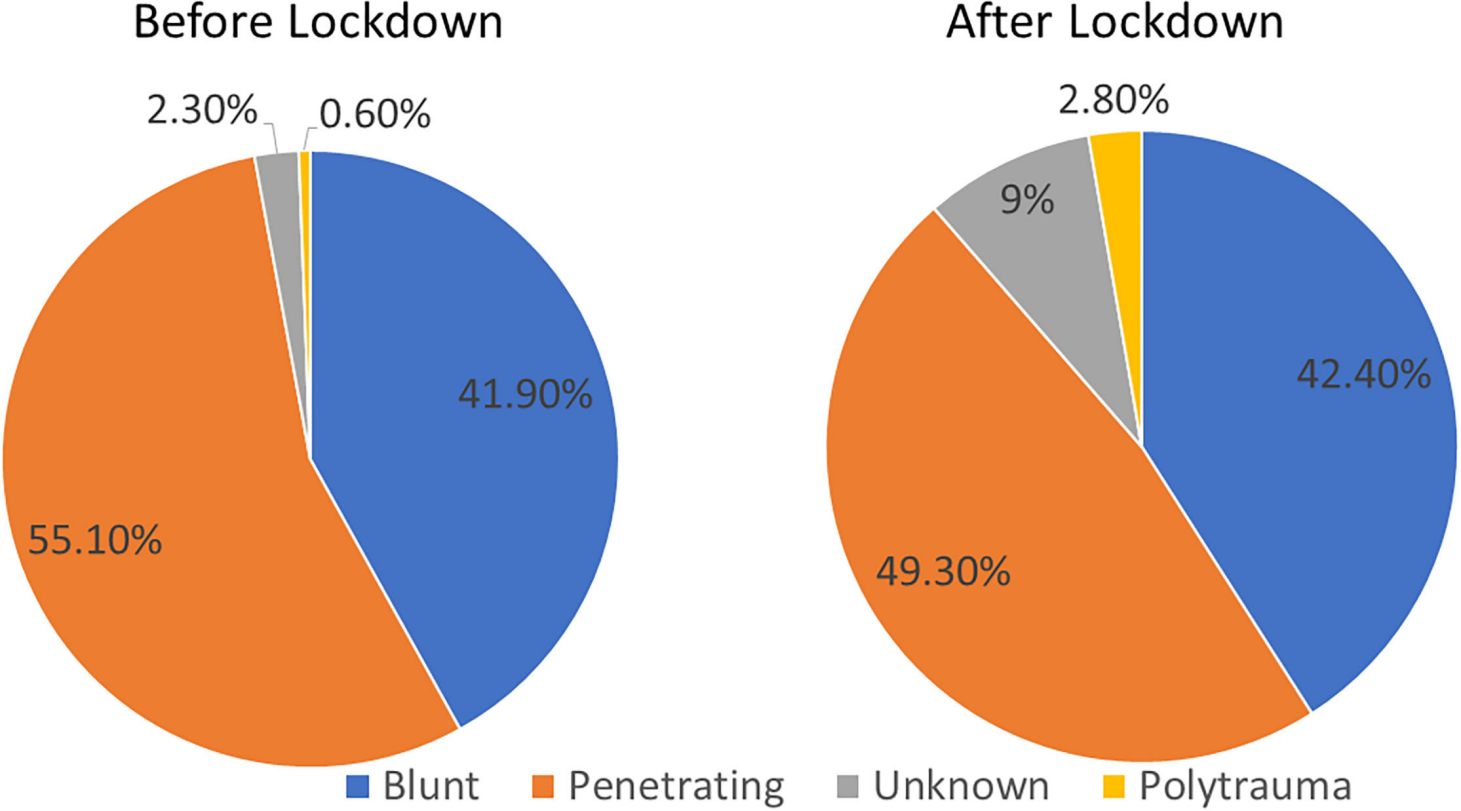
Trauma distribution for all patients before lockdown and after lockdown.

**Figure 2:**
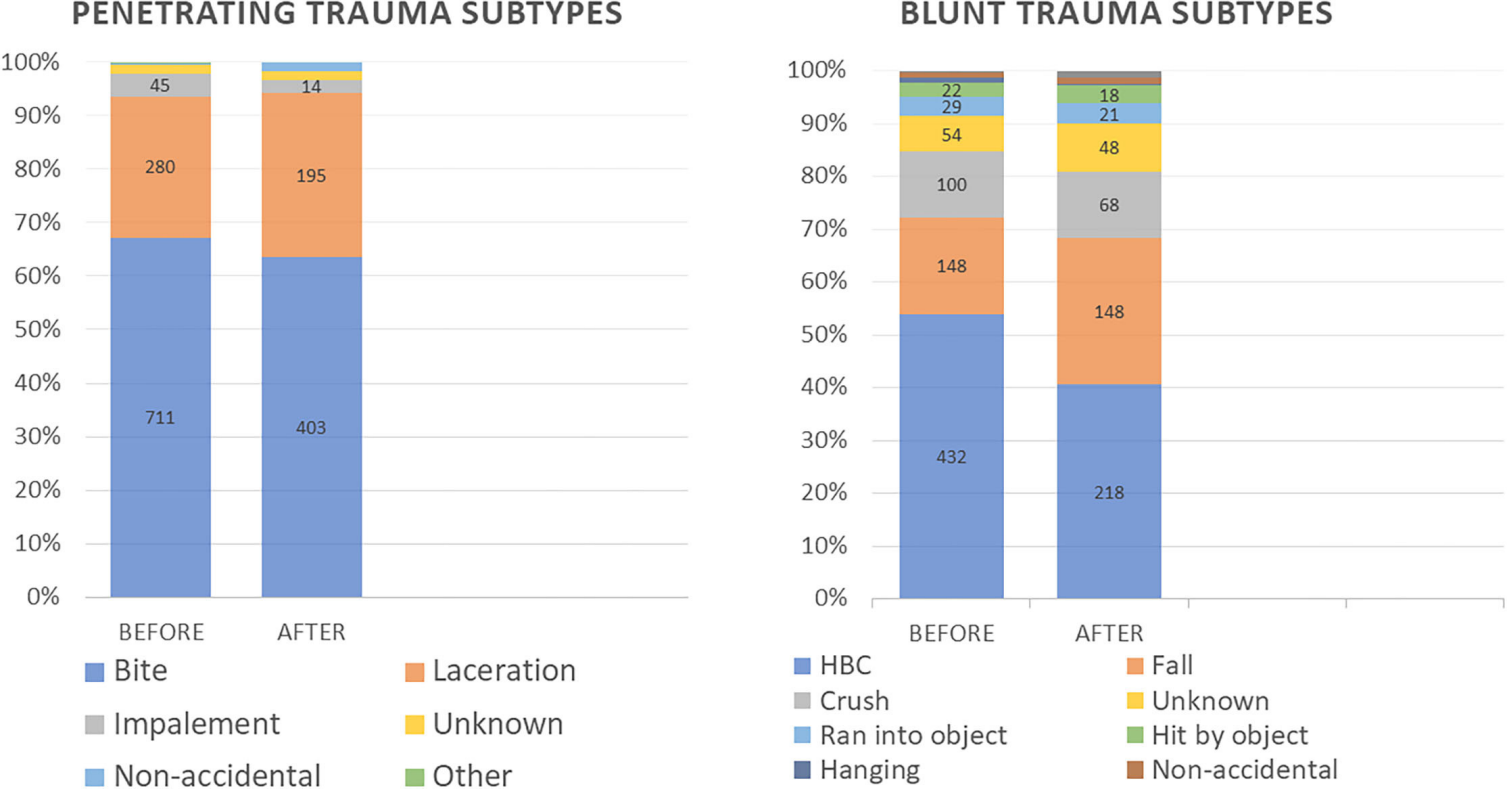
Penetrating and Blunt Trauma subtype distribution for all patients before lockdown and after lockdown.

Proportions of trauma types significantly differed BEFORE vs. AFTER (*p* < 0.01), while penetrating trauma remained the most common (Figure 1). Distribution of subtypes of both penetrating and blunt trauma significantly changed AFTER vs. BEFORE (*p* = 0.025 and *p* = 0.028, respectively) as detailed in Figure 2.

Rates of interventions differed between time periods, with rates of surgical procedures significantly increased AFTER (*p* = 0.015). The number of patients without interventions significantly decreased AFTER compared to BEFORE (*p* = 0.039).

Survival rates significantly increased to 91% AFTER (*p* = 0.013) leading to a decrease in mortality rates from 12.2 to 9%. Just over 8% of the BEFORE population were euthanized and 4% died from CPA while 6.4% of the AFTER population were euthanized and 2.6% died from CPA.

Trauma prevalence was significantly different (*p* < 0.0001) between the time periods, with a BEFORE prevalence of 2.8% which increased to 3.1% AFTER.

### Feline vs. Canine

The proportion of feline patients to canine patients was not significantly different BEFORE vs. AFTER, with cats continuing to make up ~20% of all trauma patients included in the study (*p* = 0.595). Table 3 provides information on feline and canine patients both over the entire study as well as divided into BEFORE and AFTER. While median age was not significantly different between species and time periods, when comparing age categories there were significant differences with juvenile cats making up a higher proportion than juvenile dogs overall (*p* = 0.002). In addition, the juvenile population increased during the AFTER for dogs only (*p* = 0.032). While sex distribution and neuter status was significantly different between dogs and cats (*p* < 0.001), no significant differences were identified within each species through the time periods.

**Table 3:** Patient characteristics, interventions, and outcomes by species.

**Species:**	**Cat**				**Dog**				
**Time period:**	**Overall**	**BEFORE**	**AFTER**		**Overall**	**BEFORE**	**AFTER**		
	**Count (% of total) or Median (IQR)**				**Count (% of total) or Median (IQR)**				
				Significance^*^between time periods per species (*P*-values ≤ 0.05 are in *bold italics*)				Significance^*^between time periods per species (*P*-values ≤ 0.05 are in *bold italics*)	Significance^*^between species (*P*-values ≤ 0.05 are in *bold italics*)
**Total count**	**682**	**416**	**266**		**2,507**	**1,501**	**1,006**		
**Patient signalment**		
*Age*	3 (6)	3 (6)	3 (6.075)	*P* = 0.395	4 (7)	4 (6)	4 (6.5)	*P* = 0.069	*P* = 0.069
*Age group*				*P* = 0.714				***P = 0.032***	***P = 0.002***
Juvenile	164 (24%)	98 (23.6%)	66 (24.8%)		471 (18.9%)	261 (17.4%)	210 (20.9%)		
Adult	518 (7%)	318 (76.4%)	200 (75.2%)		2,036 (81.1%)	1,240 (82.6%)	796 (79.1%)		
*Sex & desexing status*				*P* = 0.127				*P* = 0.403	***P < 0.001***
Male entire	86 (12.6%)	59 (14.2%)	27 (10.2%)		486 (19.4%)	291 (19.4%)	195 (19.4%)		
Male desexed	294 (43.1%)	176 (42.3%)	118 (44.4%)		832 (33.2%)	513 (34.2%)	319 (31.7%)		
Male unreported	1 (0.1%)	0 (0%)	1 (0.4%)		1 (<0.1%)	0 (0%)	1 (0.1%)		
Female entire	75 (11%)	52 (12.5%)	23 (8.6%)		374 (14.9%)	218 (14.5%)	156 (15.5%)		
Female desexed	220 (32.2%)	125 (30%)	95 (35.7%)		801 (32%)	473 (31.5%)	328 (32.6%)		
Female unreported	1 (0.1%)	0 (0%)	1 (0.4%)		7 (0.3%)	2 (0.1%)	5 (0.5%)		
Unknown	5 (0.7%)	4 (1%)	1 (0.4%)		6 (0.2%)	4 (0.3%)	2 (0.2%)		
**Trauma type**				***P < 0.001***				***P < 0.001***	***P < 0.001***
Penetrating	225 (33%)	151 (36.3%)	74 (27.8%)		1,459 (58.2%)	906 (60.4%)	553 (55%)		
Blunt	395 (57.9%)	243 (58.4%)	152 (57.1%)		945 (37.7%)	561 (37.4%)	384 (38.2%)		
Unknown	41 (6%)	15 (3.6%)	26 (9.8%)		77 (3.1%)	29 (1.9%)	48 (4.8%)		
Polytrauma	21 (3.1%)	7 (1.7%)	14 (5.3%)		26 (1%)	5 (0.3%)	21 (2.1%)		
**Trauma subtypes**	
*Penetrating subtypes*				*P* = 0.361				***P = 0.002***	***P < 0.001***
Bite	135 (19.8%)	93 (22.4%)	42 (15.8%)		979 (39.1%)	618 (41.2%)	361 (35.9%)		
Impalement	7 (1%)	6 (1.4%)	1 (0.4%)		52 (2.1%)	39 (2.6%)	13 (1.3%)		
Laceration	68 (10%)	41 (9.9%)	27 (10.2%)		407 (16.2%)	239 (15.9%)	168 (13.7%)		
Non-accidental	1 (0.1%)	0 (0%)	1 (0.4%)		12 (0.5%)	2 (0.1%)	10 (1%)		
Unknown	20 (3%)	12 (2.9%)	8 (3%)		7 (0.3%)	4 (0.3%)	3 (0.3%)		
Other	1 (0.1%)	1 (0.2%)	0 (0%)		3 (0.1%)	3 (0.2%)	0 (0%)		
*Blunt subtypes*	395 (57.9%)	243 (58.4%)	152 (57.1%)	*P* = 0.174	945 (37.7%)	561 (37.4%)	384 (38.2%)	***P = 0.002***	***P < 0.001***
HBC	164 (24%)	113 (27.2%)	51 (19.2%)		486 (19.4%)	319 (21.3%)	167 (16.6%)		
Fall	85 (12.5%)	43 (10.3%)	42 (15.8%)		211 (8.4%)	105 (7%)	106 (10.5%)		
Crush	60 (8.8%)	38 (9.1%)	22 (8.3%)		108 (4.3%)	62 (4.1%)	46 (4.6%)		
Ran into object	7 (1%)	3 (0.7%)	4 (1.5%)		43 (1.7%)	26 (1.7%)	17 (1.7%)		
Struck by object	4 (0.6%)	3 (0.7%)	1 (0.4%)		36 (1.4%)	19 (1.3%)	17 (1.7%)		
Non-accidental	4 (0.6%)	2 (0.5%)	2 (0.8%)		9 (0.4%)	5 (0.3%)	4 (0.4%)		
Unknown	63 (9.2%)	36 (8.7%)	27 (10.2%)		39 (1.6%)	18 (1.2%)	21 (2.1%)		
Hanging	4 (0.6%)	3 (0.7%	1 (0.4%)		6 (0.2%)	5 (0.3%)	1 (0.1%)		
Other	3 (0.4%)	1 (0.2%)	2 (0.8%)		7 (0.3%)	2 (0.1%)	5 (0.5%)		
**Interventions**	
None	466 (68.3%)	283 (68%)	183 (68.8%)	*P* = 0.540	1,981 (79%)	1,207 (80.4%)	774 (76.9%)	***P = 0.019***	***P = 0.022***
Oxygen	51 (7.5%)	29 (7%)	22 (8.3%)	*P* = 0.553	109 (4.3%)	71 (4.7%)	38 (3.8%)	*P* = 0.273	***P = 0.001***
Transfusion	10 (1.5%)	9 (2.2%)	1 (0.4%)	*P* = 0.098	14 (0.6%)	7 (0.4%)	7 (0.7%)	*P* = 0.586	***P = 0.015***
Surgical procedure	64 (9.4%)	38 (9.1%)	26 (9.8%)	*P* = 0.789	273 (10.9%)	153 (10.2%)	120 (11.9%)	*P* = 0.191	*P* = 0.292
Mechanical ventilation	2 (0.3%)	1 (0.2%)	1 (0.4%)	*P* = 1.00	2 (<0.1%)	1 (<0.1%)	1 (0.1%)	*P* = 1.00	*P* = 0.202
*Outcome*				*P* = 0.075				*P* = 0.130	***P < 0.001***
Survival	529 (77.6%	312 (75%)	217 (81.6%)		2,312 (92.2%)	1,371 (91.3%)	941 (93.5%)		
Mortality	153 (22.4%)	104 (25%)	49 (18.4%)		195 (7.8%)	130 (8.7%)	65 (6.5%)		
Euthanasia	103 (15.1%)	67 (16.1%)	36 (13.5%)		136 (5.4%)	91 (6.1%)	45 (4.5%)		
Cardiopulmonary arrest	50 (7.3%)	37 (8.9%)	13 (4.9%)		59 (2.4%)	39 (2.6%)	20 (2%)		
**Reason for euthanasia**	**Count (% of total euthanasia)**			*P* = 0.507	**Count (% of total euthanasia)**			***P = 0.020***	*P* = 0.588
Poor prognosis	61 (59.2%)	45 (67.2%)	16 (44.4%)		66 (2.6%)	42 (46.1%)	24 (53.3%)		
Financial	25 (24.3%)	16 (23.9%)	9 (25%)		33 (1.3%)	25 (27.5%)	8 (17.8%)		
Both financial & prognosis	21 (20.4%)	13 (19.4%)	8 (22.2%)		30 (1.2%)	24 (26.4%)	6 (13.3%)		
Unreported	19 (18.4%)	11 (16.4%)	8 (22.2%)		19 (0.8%)	7 (7.7%)	12 (16.7%)		
Other	0 (0%)	0 (0%)	0 (0%)		2 (<0.1%)	1 (.1%)	1 (2.2%)		

*Adult, patients ≥ 1 year of age; AFTER, 1 year period including the first day of local hospital lockdown; BEFORE, 2-year time period prior to local hospital lockdown; HBC, hit by vehicle; Juvenile, patients < 1 year of age; Overall, entire 3 year of study*.

* *Significance via Chi Square or Fischer's Exact Test*.

**Figure 3:**
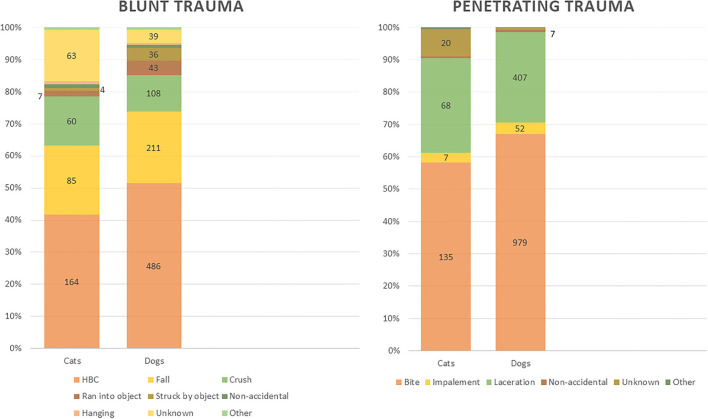
Penetrating and Blunt Trauma subtype distribution of cats and dogs for all time periods.

Overall types of trauma significantly differed between the species (*p* < 0.001) with cats having blunt trauma as the most common cause of injury (57.9%) and dogs having penetrating trauma most commonly (58.2%). Cats had significant changes (*p* < 0.001) in trauma categories BEFORE (58.4% blunt, 36.3% penetrating, 3.6% unknown, 1.7% polytrauma) vs. AFTER (57.1% blunt, 27.8% penetrating, 9.8% unknown, 5.3% polytrauma). Canine patients also had significant differences (*p* < 0.001) in trauma BEFORE (60.4% penetrating, 37.4% blunt, 1.9% unknown, 0.3% polytrauma) and AFTER (55% penetrating, 38.2% blunt, 4.8% unknown, 2.1% polytrauma). When trauma subtypes were examined between the time periods, only canine patients showed a significant difference (*p* = 0.002) (Figure 3).

The frequency of interventions differed between species with the proportion of patients receiving no interventions being higher for dogs than cats (79% and 68.3% respective, *p* = 0.022). Cats had higher prevalence of oxygen administration (7.5% cats, 4.3% dogs, *p* = 0.001) and transfusions (1.5% cats, 0.6% dogs, *p* = 0.015); there was no significant difference in the prevalence of surgical procedures (9.4% cats, 10.9% dogs, *p* = 0.292) or mechanical ventilation (0.3% cats, 0.08% dogs, *p* = 0.202) between the species.

Survival rates for cats were lower than dogs through the 3-year period (77.6% cats, 92.2% dogs, *p* < 0.001), with 15.1% of cats and 5.4% of dogs euthanized and 7.3% of cats and 2.4% of dogs dying from CPA. Reported reasons for euthanasia did not differ between species (*p* = 0.59) with poor prognosis listed as the most frequent reason (48.4% cats, 44% dogs). When comparing species across time periods, there was a significant difference in reasons for euthanasia for dogs only, with a decrease in financial or financial/prognosis reasons for euthanasia (*p* = 0.02).

### Juvenile vs. Adult

Cats and dogs <1 year of age made up 18.7% of patients BEFORE and 21.7% AFTER but this difference was not significant (*p* = 0.595). Table 4 outlines differences between age groups over the entire study period as well as between time periods. Species distribution was significantly different between age groups across the 3 years of the study. The majority of juvenile patients were dogs (74.2% dogs, 25.8% cats), as was most of the adult population (79.7% dogs, 25.8% cats) (*p* = 0.002). Not surprisingly, median age between groups was significantly different but this was not significant when time periods per age group were compared. Sex distribution and neuter status was significantly different between juveniles and adults (*p* < 0.001), with 33.7% of juvenile patients being entire males compared to 14.0% of adult patients, while 14.3% of juveniles were entire females compared to 36.4% of adult patients. When comparing age groups through time periods, there was a significant difference (*p* = 0.01) in sex and reproductive status for adult patients with the BEFORE population containing 14.2% entire males and 10.7% entire females while the AFTER population contained 13.8% entire males and 7.6% entire females.

**Table 4:** Patient characteristics, interventions, and outcomes by age groups.

**Age group:**	**Juvenile**				**Adult**				
**Time period:**	**Overall**	**BEFORE**	**AFTER**		**Overall**	**BEFORE**	**AFTER**		
	**Count (% of total) or Median (IQR)**				**Count (% of total) or Median (IQR)**				
				Significance^*^between time periods by age groups (*P*-values ≤ 0.05 are in *bold italics*)				Significance^*^between time periods by age groups (*P*- values ≤ 0.05 are in *bold italics*)	Significance^*^between age groups (*P*-values ≤ 0.05 are in *bold italics*)
**Total count**	**635**	**359**	**276**		**2,554**	**1,558**	**996**		
**Patient signalment**	
*Species*				*P* = 0.361				*P* = 0.880	***P = 0.002***
Feline	164 (25.8%)	98 (27.3%)	66 (23.9%)		518 (20.3%)	318 (20.4%)	200 (20.1%)		
Canine	471 (74.2%)	261 (72.7%)	210 (76.1)		2,036 (79.7%)	1,240 (79.6%)	796 (79.9%)		
*Age*	0.5 (0.4)	0.5 (0.4)	0.5 (0.4)	*P* = 0.452	5 (6)	5 (6)	5 (7)	*P* = 0.110	***P < 0.001***
*Sex & desexing status*				*P* = 0.258				**P = 0.010**	***P < 0.001***
Male entire	214 (33.7%)	129 (35.9%)	85 (30.8%)		358 (14%)	221 (14.2%)	137 (13.8%)		
Male desexed	120 (18.9%)	69 (19.2%)	51 (18.5%)		1,006 (39.4%)	620 (39.8%)	386 (38.8%)		
Male unreported	0 (0%)	0 (0%)	0 (0%)		2 (<0.1%)	0 (0%)	2 (0.2%)		
Female entire	207 (32.6%)	104 (29%)	103 (37.3%)		242 (9.5%)	166 (10.7%)	76 (7.6%)		
Female desexed	91 (14.3%)	55 (15.3%)	36 (13%)		930 (36.4%)	543 (34.9%)	387 (38.9%)		
Female unreported	0 (0%)	0 (0%)	0 (0%)		8 (0.3%)	2 (0.1%)	6 (0.6%)		
Unknown	3 (0.5%)	2 (0.6%)	1 (0.4%)		8 (0.3%)	6 (0.4%)	2 (0.2%)		
**Trauma type**				*P* = 0.156				***P < 0.001***	***P < 0.001***
Penetrating	220 (34.6%)	33 (9.2%)	87 (31.5%)		1,464 (57.3%)	924 (59.3%)	540 (54.2%)		
Blunt	393 (61.9%)	218 (60.7%)	175 (63.4%)		947 (37.1%)	586 (37.6%)	361 (36.2%)		
Unknown	16 (2.5%)	6 (1.7%)	10 (3.6%)		102 (4%)	38 (2.4%)	64 (6.4%)		
Polytrauma	6 (0.9%)	2 (0.6%)	4 (1.4%)		41 (1.6%)	10 (0.6%)	31 (3.1%)		
**Trauma subtypes**	
*Penetrating subtypes*				*P* = 0.277				***P = 0.003***	*P* = 0.872
Bite	146 (23%)	92 (25.6%)	54 (19.6%)		968 (37.9%)	619 (39.7%)	349 (35%)		
Impalement	8 (1.3%)	5 (1.4%)	3 (1.1%)		51 (2%)	40 (2.6%)	11 (1.1%)		
Laceration	60 (9.4%)	34 (9.5%)	26 (9.4%)		415 (16.2%)	246 (15.8%)	169 (17%)		
Non-accidental	3 (0.5%)	0 (0%)	3 (1.1%)		10 (0.4%)	2 (0.1%)	8 (0.8%)		
Unknown	3 (0.5%)	1 (0.3%)	2 (0.7%)		24 (0.9%)	15 (0.9%)	9 (0.9%)		
Other	1 (0.2%)	1 (0.3%)	0 (0%)		3 (0.1%)	3 (0.2%)	0 (0%)		
*Blunt subtypes*				***P = 0.018***				***P = 0.029***	***P < 0.001***
HBC	99 (15.6%)	65 (18.1%)	34 (12.3%)		551 (21.6%)	367 (23.6%)	184 (18.5%)		
Fall	142 (22.4%)	63 (17.5%)	79 (28.6%)		154 (6%)	85 (5.5%)	69 (6.9%)		
Crush	85 (13.4%)	53 (14.8%)	32 (11.6%)		83 (3.2%)	47 (3%)	36 (3.6%)		
Ran into object	17 (2.7%)	8 (2.2%)	9 (3.3%)		33 (1.3%)	21 (1.3%)	12 (1.2%)		
Struck by object	10 (1.6%)	7 (1.9%)	3 (1.1%)		30 (1.2%)	15 (1%)	15 (1.5%)		
Non-accidental	5 (0.8%)	2 (0.6%)	3 (1.1%)		8 (0.3%)	5 (0.3%)	3 (0.3%)		
Unknown	28 (4.4%)	16 (4.5%)	12 (4.3%)		74 (2.9%)	38 (2.4%)	36 (3.6%)		
Hanging	5 (0.8%)	4 (1.1%)	1 (0.4%)		5 (0.2%)	4 (0.3%)	1 (0.1%)		
Other	2 (0.3%)	0 (0%)	2 (0.7%)		8 (0.3%)	3 (0.3%)	5 (0.5%)		
**Interventions**	
None	474 (74.6%)	271 (75.5%)	203 (73.6%)	*P* = 0.669	1,973 (77.3%)	1,219 (78.2%)	754 (75.7%)	***P = 0.042***	*P* = 0.728
Oxygen	43 (6.8%)	29 (8.1%)	14 (5.1%)	*P* = 0.153	117 (4.6%)	71 (4.6%)	46 (4.6%)	*P* = 1.00	***P = 0.032***
Transfusion	6 (0.9%)	3 (0.8%)	3 (1.1%)	*P* = 1.00	18 (0.7%)	13 (0.8%)	5 (0.5%)	*P* = 0.468	*P* = 0.606
Surgical procedure	67 (10.6%)	38 (10.6%)	29 (10.5%)	*P* = 1.00	270 (10.6%)	153 (9.8%)	117 (11.7%)	*P* = 0.129	*P* = 1.00
Mechanical ventilation	1 (0.1%)	1 (0.3%)	0 (0%)	*P* = 1.00	3 (0.1%)	1 (<0.1%)	2 (0.2%)	*P* = 0.564	*P* = 0.589
**Outcome**				*P* = 0.759				***P = 0.020***	***P = 0.001***
Survival	588 (92.6%)	330 (91.9%)	258 (93.5%)		2,253 (88.2)	1,353 (86.8%)	900 (90.4%)		
Total mortality	47 (7.4%)	29 (8.1%)	18 (6.5%)		301 (11.8%)	205 (13.1%)	96 (9.6%)		
Euthanasia	26 (4.1%)	16 (4.5%)	10 (3.6%)		213 (8.3%)	142 (9.1)	71 (7.1%)		
Cardiopulmonary arrest	21 (3.3%)	13 (3.6%)	8(2.9%)		88 (3.4%)	63 (4%)	25 (2.5%)		
**Reason for euthanasia**	**Count (% of total euthanasia)**			*P* = 0.715	**Count (% of total euthanasia)**			***P = 0.045***	*P* = 0.244
Poor prognosis	15 (57.7%)	8 (50%)	7 (70%)		112 (52.6%)	79 (55.6%)	33 (46.5%)		
Financial	10 (38.5%)	7 (43.8%)	3 (30%)		48 (22.5%)	34 (23.9)	14 (19.7%)		
Both financial & prognosis	8 (30.8%)	5 (31.3%)	0 (0%)		43 (20.2%)	32 (22.5%)	11 (15.5%)		
Unreported	1 (3.8%)	1 (6.3%)	0 (0%)		37 (17.4%)	17 (12%)	20 (28.2%)		
Other	0 (0%)	0	0 (0%)		2 (0.9%)	1 (0.7%)	1 (1.4%)		

*Adult, patients ≥ 1 year of age; AFTER, 1 year period including the first day of local hospital lockdown; BEFORE, 2-year time period prior to local hospital lockdown; HBC, hit by vehicle; Juvenile, patients < 1 year of age; Overall, entire 3 year of study*.

* *Significance via Chi Square or Fischer's Exact Test*.

**Figure 4:**
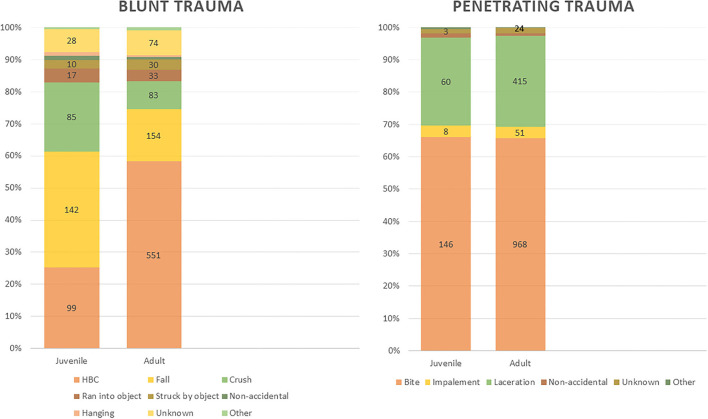
Penetrating and Blunt Trauma subtype distribution of juvenile and adult patients for all time periods.

Overall types of trauma significantly differed between the age groups (*p* < 0.001) with blunt trauma being the most common form of juvenile trauma (61.9%) and penetrating trauma the most common in adult patients (57.3%). Comparing age groups between time periods found changes in trauma categories only significantly (*p* < 0.001) affecting adult patients with BEFORE patterns including 59.3% penetrating, 37.6% blunt, 2.4% unknown, and 0.6% polytrauma while AFTER patterns were 54.2% penetrating, 36.2% blunt, 6.4% unknown, and 3.1% polytrauma. When trauma subtypes were examined, significant differences were identified both overall and when comparing age groups between time periods (Figure 4).

The frequency of interventions differed between age groups with juveniles having increased rates of oxygen administration (6.8% juvenile, 4.6% adult, *p* = 0.032); there was no difference in the rates of transfusions, surgical procedures, or mechanical ventilation between the age groups.

Survival rates for juveniles were significantly higher than adults (92.6% juvenile, 88.2% adult, *p* = 0.001) through the 3-year period. Similarly, there was a difference in euthanasia rates (4.1% juvenile, 8.3% adult) but not in the occurrence of CPA (3.3% juvenile, 3.4% adult). Adult patients had differences in survival and death rates between time periods with 86.8% surviving, 9.1% being euthanized and 4% suffering CPA BEFORE while 90.4% survived, 7.1% were euthanized and 2.5% suffering CPA AFTER (*p* = 0.02). Reported reasons for euthanasia did not differ between age groups with poor prognosis as the most frequently reported reason (44.1% juvenile, 46.3% adult, *p* = 0.24). Adult patients also showed a significant difference between time periods with decreases in all reported reasons except for unreported and other categories (*p* = 0.045).

## Discussion

Trauma prevalence has been reported in varying percentages and permutations. They vary from 2.7% of dogs in a group of small animal primary care practices in Korea and 4.6% of cats in multiple veterinary practices in the UK, to 11.4% of all dogs at government and private practice hospitals in Nigeria and 13% of all dogs and cats at an urban referral hospital in the USA (19–22). The current study, focused on referral hospitals in an urban and suburban environment in Australia, identified a relatively low trauma prevalence which significantly increased follow the onset of lockdown. Pre-lockdown trauma patterns in both species in this study closely mirrored those patterns reported in other studies, with cats more likely to experience blunt trauma and dogs, penetrating trauma (1–8). However, the distribution of trauma categories changed once lockdown started, with an overall decrease in penetrating injuries and an increase in unknown trauma. These findings parallel that which has been reported in the human literature, where the social restrictions of lockdown have led to a change in the pattern and number of trauma presentations but not in prevalence (13, 14). Reasons for the changes in overall trauma distribution and trauma prevalence are not clear. Possible factors include changes in human interactions with animals as well as interactions between animals.

When trauma subtypes were examined, there were no apparent differences in the types of penetrating traumas between time periods. However, in the category of blunt trauma, there was a decrease in the proportion of vehicular related traumas but an increase in the proportion of falls. This change was across species. The former is likely to be due to societal restrictions during lockdown; there was a significant decrease in travel by the population for work and recreation which translated into less vehicles on the road (23, 24). This was a similar finding in the human literature (13, 14). In contrast, an increase in trauma at home is expected due to increased human-animal interaction for prolonged periods of time in potentially confined spaces. For example, children playing with and chasing their pet dogs in a small apartment may lead to accidental injury and falls. This increase in certain subtypes of trauma paired with an increase in pet adoptions may be the reason for the increase in trauma prevalence post-lockdown (25).

When comparing trauma subtypes between cats and dogs, significant differences were identified. With blunt trauma, cats had a lower proportion of HBC than dogs and higher proportions of unknown trauma. Similarly, with penetrating trauma, cats had less bite wounds but more unknown trauma than dogs. While the trauma subtypes changed within species when comparing time periods, only canine trauma subtype changes were significant. This may be due to the canine population making up the majority of the study population, resulting in a smaller feline population unable to reflect significant differences. Similar to reasons for the overall change in trauma subtypes with combined species, the locations where trauma occurred (at home vs. outside of the home) could have been affected by changes in human movement.

Juvenile patients of both species had trauma patterns that were closer to feline trauma pattern than canine patterns, with blunt trauma being most common when compared to adults of both species. This comparison continues when evaluating the blunt trauma subtypes, with juveniles having much higher rates of falls and crush injury; for adult patients, HBC is the most common blunt trauma subtype. Interestingly, penetrating trauma subtypes did not differ significantly between age groups. While many studies have investigated correlations of trauma with age, no major studies have focused on differences in trauma patterns between juvenile and adult patients. One study (26) identified that juvenile dogs had higher rates of death from trauma when compared to adult dogs while another study (27) showed that risks of road traffic accidents were highest for juvenile cats when compared to adult cats. The differences in trauma patterns and trauma subtypes identified in the current study are likely due to restricted roaming of juveniles, as they are less likely to be out of the house or yard, or walked off lead, while adults are more likely to either escape or roam freely, increasing their interaction with vehicles and risk of HBC trauma.

This study supports anecdotal reports of an increase in puppy ownership during lockdowns (15, 16). However, a similar increase in kittens presenting for trauma during lockdown was not evident. This may be due to an increase in feline ownership occurring across all age ranges, a decreased likelihood for kittens to experience trauma compared to puppies or the relatively small feline population leading to a Type II error. The juvenile population showed lower rates of desexing during lockdown when compared to pre-lockdown. This may be a reflection of the lack of availability for desexing at veterinary clinics or a knowledge deficiency of owners with an increase in new pet ownership (15, 16).

While there was a concern that owner finances would contribute to poorer outcomes and reduced interventions due to job losses during COVID, this was not supported by the study results. Fewer patients in the post-lockdown group received no intervention. Among interventions, surgical procedures significantly increased AFTER while oxygen therapy, blood transfusions and mechanical ventilation did not. When comparing rates of interventions by species and age groups, there are significant differences. The number of cats not receiving interventions was lower than dogs overall. While no significant difference for this was found with cats between time periods, the number of dogs not receiving interventions decreased AFTER. Over both time periods, cats were more likely to receive oxygen supplementation and blood transfusions than dogs. No difference in type of interventions was found with cats between time periods while dogs AFTER had decreases in rates of oxygen administration but increases in blood transfusions. The number of juvenile patients not having interventions was statistically unchanged between time periods. Adult patients without interventions decreased in number AFTER. Juvenile patients were significantly more likely to get oxygen supplementation than adults at all time periods. No differences were found in the rates of individual interventions by age group between time periods. It is possible that lockdowns have led to a strengthening of the human-animal bond, resulting in an increase in presentations to emergency and a greater willingness to pursue interventions aimed at improving survival. The financial effect of pet insurance might also have played a role, but information about the frequency of pet insurance was not obtainable due to the retrospective nature of this study. The relatively low numbers of interventions across both time periods indicates that this study included patients with mild trauma, which makes up the majority of trauma patients, as well as more severe trauma, which follows a pattern previously reported (5). Less expensive interventions were not included in this study as they were deemed unlikely to be affected by any negative financial effects from lockdown. This may have led to bias and including information on less expensive interventions may have given more insight to financial effects which were not evident with the selected major interventions.

Despite an increase in trauma prevalence AFTER, survival rates increased significantly from 87.8 to 91%. With the social changes during lockdown including pet owners spending more time with and observing their pets at home, it is likely that cases of trauma were identified earlier. Early presentation of trauma cases to emergency possibly contributed to improved outcomes and therefore survival (1, 4). Another contributing factor for increased survival is the interventions that were performed. As has been previously reported, dogs had higher survival rates than cats. While survival rates increased within the individual species between time periods, these changes were not significant, potentially due to insufficient case numbers. Reasons for the continued differences in survival rates between species include differences in body size, physiology, owner attachment, and the types of trauma experienced. Juvenile patients had a significantly higher survival rates than adults. This may be due to the nature of the age distribution, with the adult population containing animals with poorer health and increased comorbidities as well as geriatric patients and those with chronic diseases. Other reasons for higher survival rates for juveniles include improved healing ability in the young and potentially a decreased willingness for owners to euthanize a young pet. While juveniles did not have a significant increase in survival between time periods, adults had a significant improvement in survival. When the reasons for euthanasia was reported, there was no significant difference between species or age groups across time periods. While it can be difficult to elucidate the reason for euthanasia in a retrospective study, the participating hospitals commonly reported this in the medical records and the proportion of patients with an unreported reason for euthanasia was low across time periods. Dogs and adult patients had decreases in euthanasia for financial or financial and prognosis reasons AFTER, with adult patients also having a decrease in euthanasia for prognosis alone AFTER. These findings, combined with the increase in interventions, suggests that there was not a negative financial impact of care for these trauma patients. It is likely that lockdowns have led to a stronger bond between owners and their pets, resulting in an increase in presentations to emergency and interventions improving survival of trauma patients.

This study has several limitations. As a retrospective study involving multiple hospitals, treatment protocols may have differed, leading to bias. In addition, the lack of trauma scores such as Acute Trauma Triage Score or modified Glasgow Coma Scale in the medical records prevented any analysis of the effects of trauma severity on patient interventions and outcome in this population as well as identify if the changes in trauma subtypes was associated with more or less severe injuries between the time periods. Similarly, no information on hospital costs was collected, which might have correlated with the severity of injury. Behaviors leading to the patient's trauma were not recorded, so true changes in human traffic and exercise patterns could not be directly investigated between the time periods. Lastly, all patients presented to referral hospitals, which means these results may not represent a true trauma prevalence or identify trauma patterns accurately for the pet population.

Data collection occurred at hospitals in the two largest cities (SYD, MEL) and a smaller city with semirural clients (AREC); a larger sample size including a diverse range of clinics may have resulted in further statistically significant changes in prevalence between time periods. Although there have been studies in the human literature assessing trauma prior to and after lockdowns during COVID, this is the first veterinary study. Further investigation of this topic would help determine if the reported changes are universal or regional.

In conclusion, this study identified that cats and dogs have different trauma patterns, with cats more likely to suffer blunt trauma and dogs, penetrating trauma, and different survival rates (77.6% cats, 92.2% dogs). Trauma patterns changed for cats and dogs in the post-lockdown period with cats having a decrease in penetrating trauma but an increase in unknown trauma while dogs had a decrease in penetrating trauma and an increase in unknown and polytrauma. Juvenile patients also have different trauma patterns and survival rates than adults. Compared to pre-pandemic times, COVID lockdown was associated with a higher trauma prevalence but improved survival rates regardless of species or age category. A decrease in financially driven euthanasia and an increase in interventions suggest no negative financial effect of lockdown on trauma veterinary patient care in Australia.

## Author's Note

Partial results were presented at the 2021 Australia New Zealand College of Veterinary Scientists Emergency and Critical Care Chapter's virtual Science Week and at the American College of Veterinary Emergency and Critical Care Veterinary Committee on Trauma conference in March 2022.

## Data Availability Statement

The raw data supporting the conclusions of this article will be made available by the authors, without undue reservation.

## Ethics Statement

Ethical review and approval was not required for the animal study because this was a retrospective study of privately-owned animals presenting to veterinary hospitals for medical care. Written informed consent for participation was not obtained from the owners because this was a retrospective study.

## Author Contributions

MH was responsible for study design, data collection, statistical analysis, and draft writing/editing. EN and HO performed data collection, draft writing, and editing. All authors contributed to the article and approved the submitted version.

## Conflict of Interest

The authors declare that the research was conducted in the absence of any commercial or financial relationships that could be construed as a potential conflict of interest.

## Publisher's Note

All claims expressed in this article are solely those of the authors and do not necessarily represent those of their affiliated organizations, or those of the publisher, the editors and the reviewers. Any product that may be evaluated in this article, or claim that may be made by its manufacturer, is not guaranteed or endorsed by the publisher.
